# Evolutionary Trajectory of White Spot Syndrome Virus (WSSV) Genome Shrinkage during Spread in Asia

**DOI:** 10.1371/journal.pone.0013400

**Published:** 2010-10-14

**Authors:** Mark P. Zwart, Bui Thi Minh Dieu, Lia Hemerik, Just M. Vlak

**Affiliations:** 1 Laboratory of Virology, Wageningen University, Wageningen, The Netherlands; 2 Quantitative Veterinary Epidemiology Group, Wageningen University, Wageningen, The Netherlands; 3 Instituto de Biología Molecular y Celular de Plantas, Consejo Superior de Investigaciones Científicas-UPV, Valencia, Spain; 4 Biotechnological Research and Development Institute, Can Tho University, Can Tho City, Vietnam; 5 Biometris, Wageningen University, Wageningen, The Netherlands; Veterinary Laboratories Agency, United Kingdom

## Abstract

**Background:**

White spot syndrome virus (WSSV) is the sole member of the novel Nimaviridae family, and the source of major economic problems in shrimp aquaculture. WSSV appears to have rapidly spread worldwide after the first reported outbreak in the early 1990s. Genomic deletions of various sizes occur at two loci in the WSSV genome, the ORF14/15 and ORF23/24 variable regions, and these have been used as molecular markers to study patterns of viral spread over space and time. We describe the dynamics underlying the process of WSSV genome shrinkage using empirical data and a simple mathematical model.

**Methodology/Principal Findings:**

We genotyped new WSSV isolates from five Asian countries, and analyzed this information together with published data. Genome size appears to stabilize over time, and deletion size in the ORF23/24 variable region was significantly related to the time of the first WSSV outbreak in a particular country. Parameter estimates derived from fitting a simple mathematical model of genome shrinkage to the data support a geometric progression (*k*<1) of the genomic deletions, with *k* = 0.371±0.150.

**Conclusions/Significance:**

The data suggest that the rate of genome shrinkage decreases over time before attenuating. Bioassay data provided support for a link between genome size and WSSV fitness in an aquaculture setting. Differences in genomic deletions between geographic WSSV isolates suggest that WSSV spread did not follow a smooth pattern of geographic radiation, suggesting spread of WSSV over long distances by commercial activities. We discuss two hypotheses for genome shrinkage, an adaptive and a neutral one. We argue in favor of the adaptive hypothesis, given that there is support for a link between WSSV genome size and fitness.

## Introduction

White spot syndrome virus (WSSV) was first reported in shrimp aquaculture in Taiwan in the early 1990s [1]. The virus has since spread worldwide, and has had a major negative impact on shrimp aquaculture [2], [3]. WSSV is a dsDNA virus with approximately a 300 kilo base pair (kbp) genome [4], [5], having one of the largest genomes of the animal viruses [6]. The virus has been recently assigned as the sole member of a new virus family, the Nimaviridae [7].

During WSSV spread in Asia the virus has shown striking changes in biological characteristics [8] and genotype [9], [10], [11]. Relative to the putative ancestral virus, a WSSV variant that evolved in shrimp aquaculture is characterized by (i) causing higher host mortality, (ii) having a shorter host survival time, and (iii) having a higher within-host competitive fitness [8]. The most significant genotypic changes during WSSV spread in Asia appear to be two genomic deletions, in the ORF14/15 and ORF23/24 variable regions [8], [9], [10], [11], [12]. The total size of the genomic deletions which have occurred between the oldest and the most recent WSSV isolates analyzed to date is almost 15 kbp [9], [11], approximately 5% of the genome.

The remainder of the WSSV genome appears to harbor very limited variation, with the exception of variable number tandem repeat (VNTR) loci [10], [11], [13], [14], [15]. The amount of variation is so limited that standard phylogenetic approaches have been of limited value in understanding WSSV evolution and spread [11], although phylogenetic analysis of a large number of full genome sequences obtained by next generation sequencing techniques may be more informative. Deletions in the ORF14/15 and ORF23/24 variable regions have, however, been used as molecular markers to study patterns of virus spread on intermediate and large spatiotemporal scales [8], [9], [10], [11], [12]. Given that all WSSV isolates genetically characterized appear to have a very recent common ancestor, the consensus view is that during WSSV spread throughout Asia the DNA genome has been progressively shrinking [3], [8], [9], [10], [11], [12], [16], [17]. In the analysis and interpretation of data reported here, we use this perspective as a starting point. Whether all WSSV strains in aquaculture derive from the Asian outbreak has not been shown, and other sources of WSSV may very well be found in other wild populations of crustaceans [18].

Although the ORF14/15 and ORF23/24 variable regions have been employed as molecular markers in many studies, there is no framework for understanding the underlying dynamics driving molecular evolution at these loci. There is good evidence that the genomic regions in question are redundant in an aquaculture environment, and it has therefore been suggested that deletion thereof contributes to increased viral fitness [8]. Another study has even suggested that very small differences in genome size may influence within-host competitive fitness [19]. Conflicting results have been found for WSSV [17], however, and in general there is little evidence to substantiate a link between genome size and replicative fitness (e.g., [20]). In summary, based on the relevant literature we make the following three assumptions: (i) a recent common ancestor in Asia has been responsible for all spread of WSSV, (ii) genomic sequences that are redundant in an aquaculture environment have been present, and (iii) consequently the redundant sequences have been progressively removed during virus evolution.

**Figure 1:**
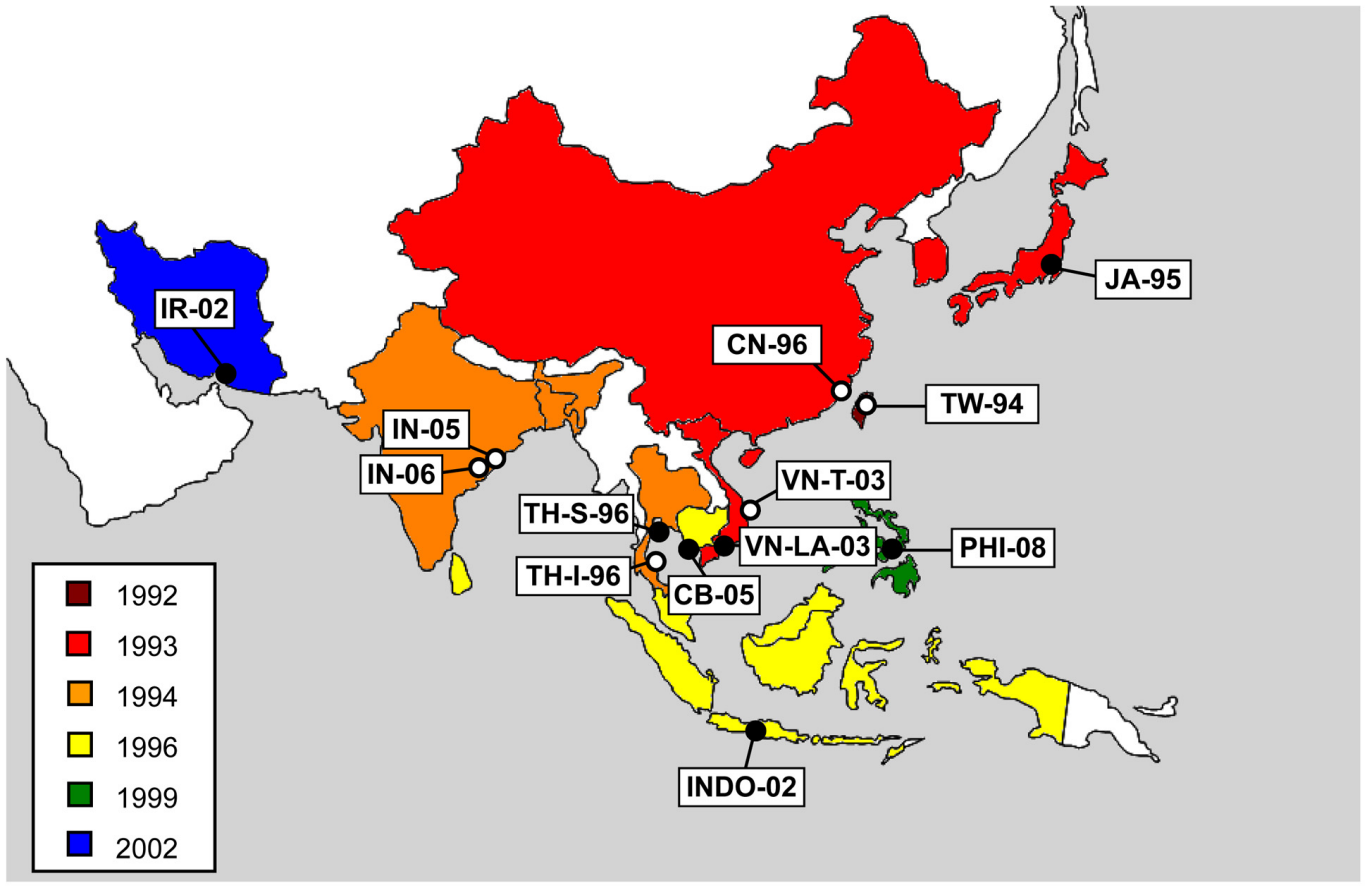
Map of Asia, showing the geographical origins of WSSV isolates used for this study. New isolates characterized in this study are marked with black circles, whereas isolates previously reported in the literature are marked with white circles. Further information on the isolates is given in Table 1. Colors denote the year of first outbreak for Asian countries for which this information is known (see Materials and Methods).

To build a framework for understanding the dynamics of WSSV genome shrinkage, we address the following unresolved questions. First, genome shrinkage can only decrease until non-redundant or essential genes and regulatory sequences are disrupted or lost. Has the limit to deletion size at the ORF14/15 and ORF23/24 variable loci been reached? We hypothesize that the limit to deletion sizes will have been reached, given the high rate of evolution typical for viruses and the time window of almost a decade. Second, the temporal pattern of WSSV outbreaks in different countries in Asia does not suggest that a smooth geographic radiation took place (Fig. 1). The virus appears to rapidly traverse large geographic distances (e.g., from Taiwan in 1992, to Thailand and India in 1994), whereas some shorter distances are slowly traversed (i.e., the first WSSV outbreak in the Philippines occurred in 1999). Does molecular evolution of WSSV recapitulate the temporal pattern of spread? We think there is good support for the idea that viruses from the Asian outbreak share a very recent common ancestor. Therefore, we hypothesize that the temporal and molecular patterns will be similar to each other. Third, does the rate of WSSV genome shrinkage change over time? Given that there are redundant sequences in the WSSV genome and that the population genetics of this pathogen allow for rapid evolution of genome size [21], what does the evolutionary trajectory look like and what mechanisms can account for it? Based on previous results [8], we first hypothesize that genome size is related to fitness, and that the deletion of redundant genomic sequences is therefore an adaptive process. Moreover, we expect that the dynamics of genome shrinkage will be concordant with theoretical predictions for the evolution of fitness [22]: thus the rate of adaptation – in this case the rate of genome shrinkage – will geometrically decrease over time. To explore these issues we have characterized novel WSSV samples from five countries in Asia, revisited the ones already published, and generated a simple model of genome shrinkage. Moreover, we performed experiments to further test whether there may be a link between genome size and viral fitness.

## Results

### Description of WSSV isolates

WSSV isolates originating from a single host were collected from ponds distributed over five different Asian countries (Fig. 1, Table 1). Among these, four isolates were derived from *Penaeus* shrimp culture ponds, and one was derived from a polychaete (Indonesian isolate; see Table 1), which are known to be WSSV carriers [23]. All isolates tested positive for the presence of WSSV using a single-step PCR, according to published procedures [10].

**Table 1:** Origins of the Asian WSSV isolates used in this study.

Country	Host	1^st^ Outbreak	Place	Origin of post larvae	Sample collection	Abbreviation
Taiwan	*P. monodon*	1992	South	ND	1994	WSSV-TW
China	*P. japonicas*	1993	Xiamen	ND	1996	WSSV-CN
Vietnam	*P. monodon*	1993	Quang Ngai	Central VN	3/2003	WSSV-VN-T
Vietnam	*P. vannamei*	1993	Long An	Central VN	2003	**WSSV-VN-LA**
Thailand	*P. monodon*	1994	Suratthan	ND	5/1996	WSSV-TH-96-I
Thailand	*P. monodon*	1994	ND	ND	1996	**WSSV-TH-S**
India	*P. monodon*	1994	Andhra Pradesh	ND	2005	WSSV-In-05
India	*P. monodon*	1994	Andhra Pradesh	ND	2006	WSSV-In-06
Philippines	*P. monodon*	1999	Iloilo	Local	4/2008	**WSSV-Phi**
Indonesia	Polychaete	1996	Jambak, Java	ND	2/2008	**WSSV-Indo**
Cambodia	*P. monodon*	1996	Shihanuk Ville	Thailand	2/2006	**WSSV-Cb**
Japan	*P. japonicas*	1993	ND	ND	1995	**WSSV-Ja**
Iran	*P. indicus*	2002	Abadan	ND	6/2002	**WSSV-Ir**

“1^st^ outbreak” is the year in which a WSSV outbreak was first reported in the country where the sample was collected. Abbreviations in bold indicate the sample was genotyped in the present study. ND stands for ‘no data’.

### Variable region ORF23/24

**Figure 2:**
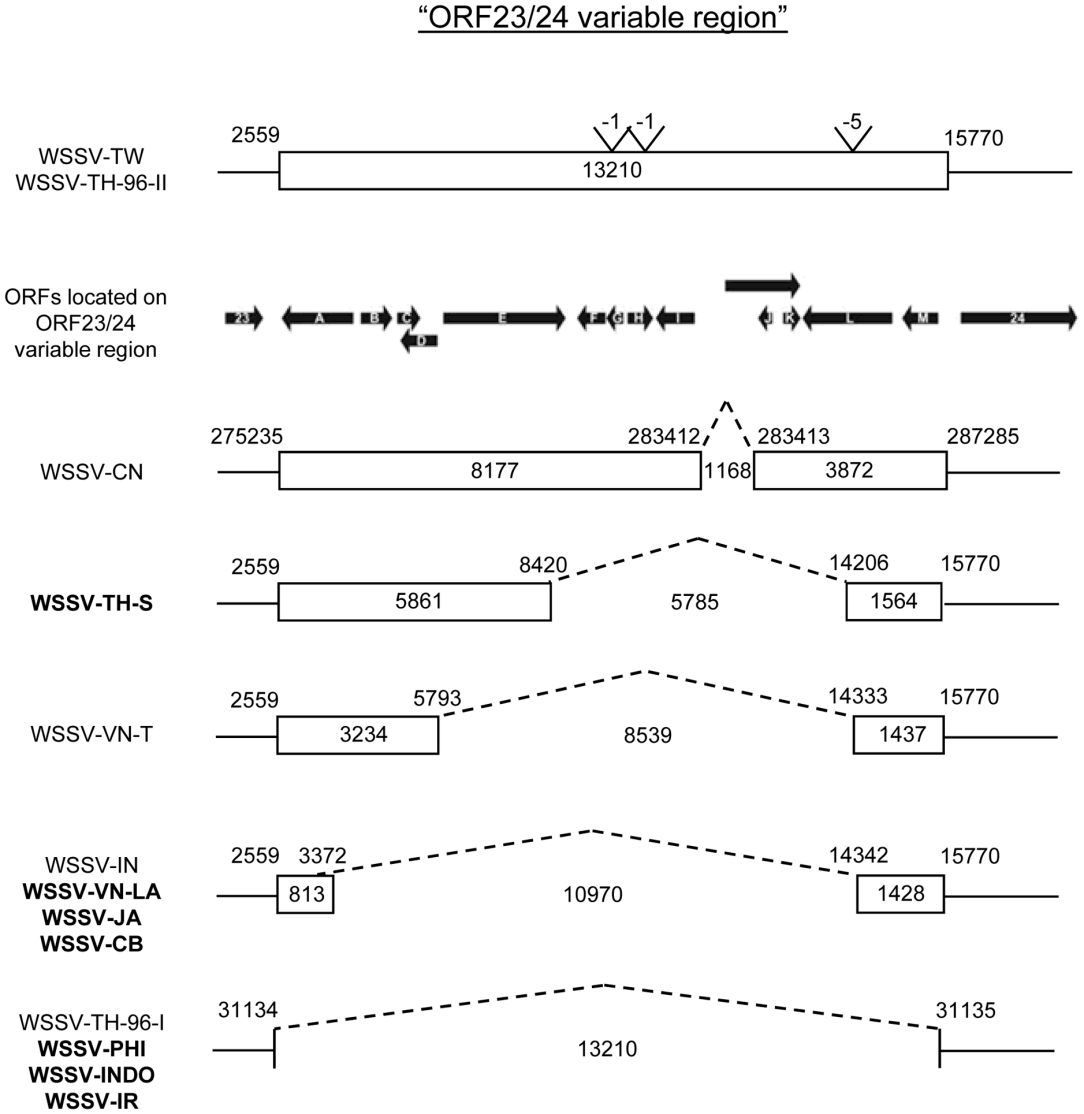
Schematic representation of the ORF23/24 variable region of the Asian WSSV isolates. The coordinates of the WSSV-Asian isolates are according to the WSSV-CN or WSSV-TH annotation. Names of WSSV isolates characterized in this study are in bold. The length of the fragments is indicated within boxes or sequences. The positions of the ORFs located in this region are indicated by closed arrows, which also represent the direction of transcription. ORFs are numbered in accordance to previous studies [8], [10].

In order to map the ORF23/24 locus, PCR with the “VR23/24-Asian screen” primers (Supplementary Table S1) was performed on all chosen samples (Fig. 2). The primer annealing sites flank the ORF23/24 variable region, based on WSSV-TH sequence (AF 369029 [4]). The WSSV isolates from the Philippines, Indonesia and Iran rendered a 400-bp amplicon. Cloning and sequencing of this PCR fragment indicated that it was 100% identical to WSSV-TH isolate, with a deletion of 13,210 bp compared to WSSV-TW. The WSSV isolates from Japan and Cambodia gave a ∼700 bp amplicon with primer set VR23/24-Ja (Table S1). Cloning and sequencing of these PCR amplicons indicated that they were 100% identical to Indian isolate (ACC. No. EU 327499 [9]), with a deletion of about 10,970 bp compared to WSSV-TW. All the characterized isolates therefore had relatively large deletions in the ORF23/24 region (Fig. 2). A Jonckheere-Terpstra test demonstrated that the ORF23/24 variable-region deletion size increased significantly with ‘first outbreak year’, but was not significantly related to ‘distance from Taiwan’ (Table 2).

**Table 2:** Statistical analysis of ORF14/15 and ORF23/24 deletion sizes of Asian WSSV isolates.

Locus	Independent variable	N	Standardized *JT*	*P* value
ORF14/15	First outbreak year	13	1.410	0.158
ORF14/15	Distance from Taiwan	13	1.040	0.298
ORF23/24	First outbreak year	13	2.848	0.004*
ORF23/24	Distance from Taiwan	13	2.051	0.040

Jonckheere-Terpstra tests were performed on the deletion size of the two variable loci. N is the number of samples, Standardized *JT* is the test statistic, and *P* value denotes the significance, which is marked with an asterisk if below the threshold *P* value of 0.025 (see Materials and Methods). First outbreak year and distance from Taiwan were not significantly correlated (Pearson correlation coefficient = 0.432, *P* = 0.141).

### Variable region ORF14/15

**Figure 3:**
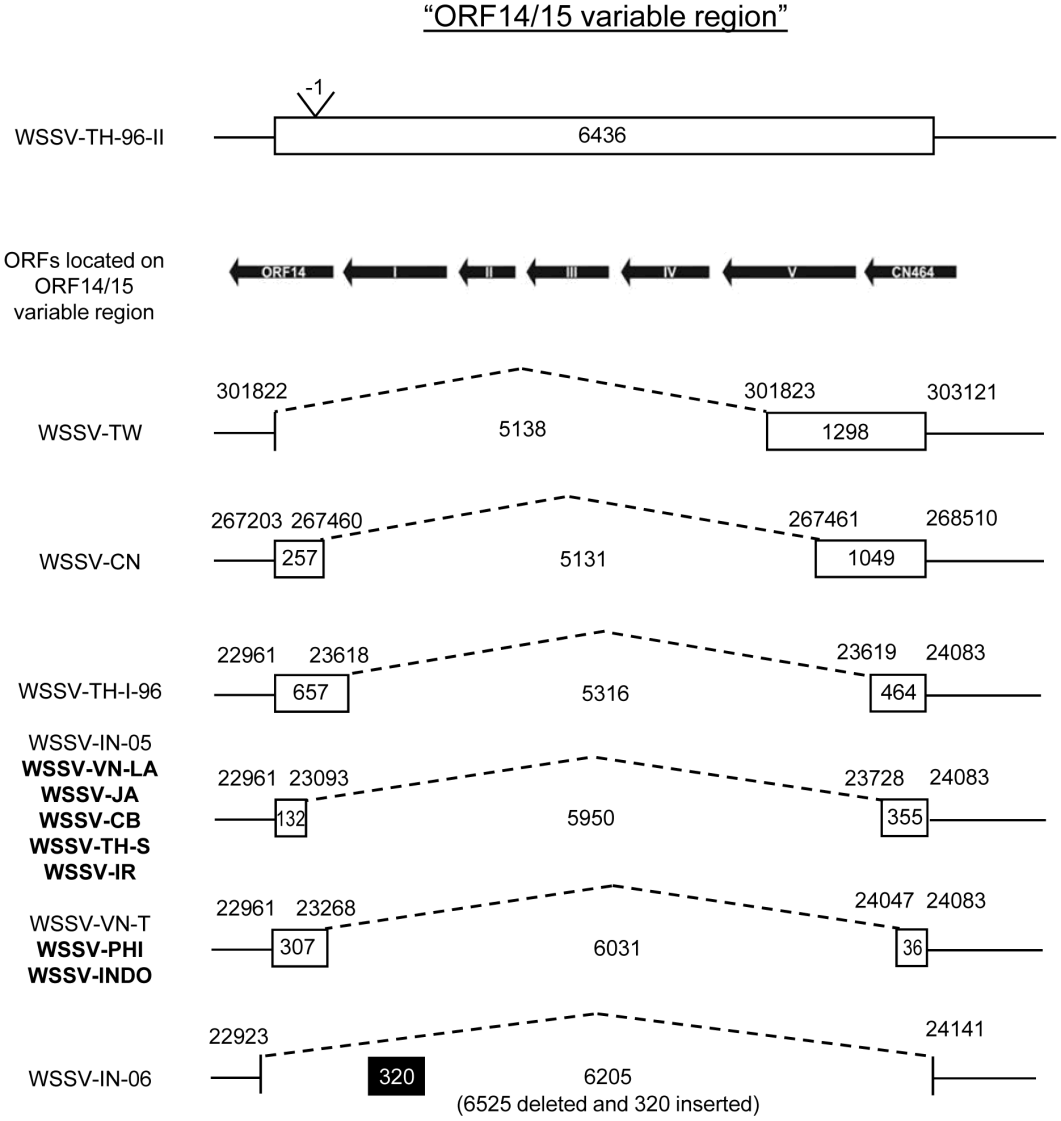
Schematic representation of the ORF14/15 variable region of the Asian WSSV isolates. Names of WSSV isolates characterized in this study are in bold. The coordinates of the WSSV-Asian isolates are given according to the WSSV TH-96-II annotation [8]. The length of the fragments is indicated within boxes or sequences. WSSV IN-06 has an inserted sequence, which is marked with a black box.

The location and size of the genomic deletion in ORF14/15 was determined for the WSSV-Asian isolates using a similar approach as for the ORF23/24 variable region. The archetype WSSV isolate TH-96-II, which has a 6,436 bp insertion compared to WSSV-TW [8], was used as a reference sequence for determining the size of the deletion. A PCR reaction with the “VR14/15-screen” primers (Table S1) was first performed. The WSSV isolates from the Philippines and Indonesia rendered a ∼500 bp amplicon, similar in length to that previously reported for isolate K from Vietnam [10]. Restriction enzyme analysis of the PCR products confirmed that these isolates have the same 6,031 bp deletion present in most WSSV-VN isolates previously analyzed. The isolates from Japan, Iran and Cambodia gave a ∼600 bp amplicon. Restriction enzyme analysis of the PCR products confirmed that these isolates have the same 5,950 bp deletion present in VN-X and VN-S (Fig. 3, [10]). A Jonckheere-Terpstra test demonstrated that the ORF14/15 variable-region deletion size was not significantly related to ‘first outbreak year’ or ‘distance from Taiwan’ (Table 2).

### Model of WSSV genome shrinkage

**Figure 4:**
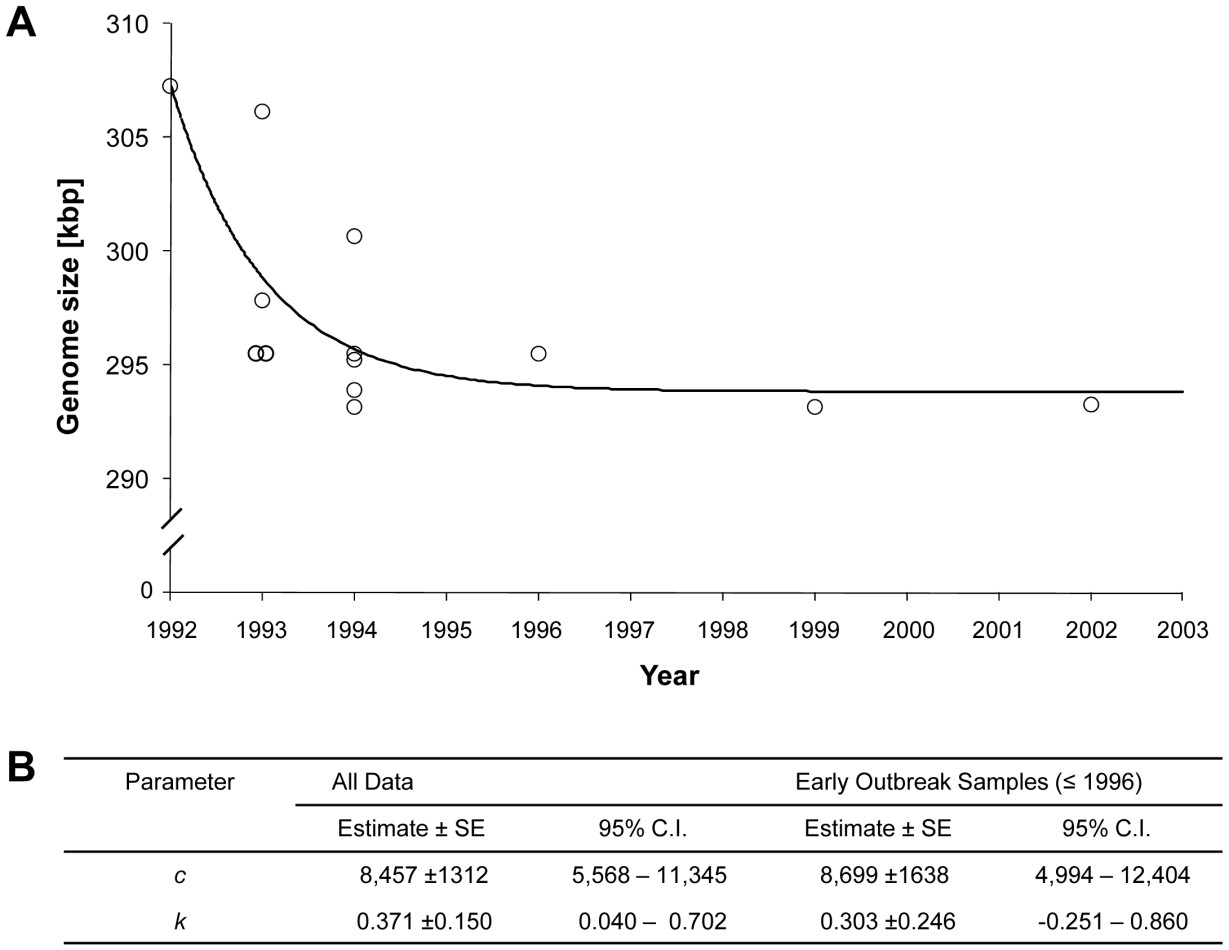
Dynamics of WSSV genome shrinkage. Panel A: the year of first outbreak for the country a WSSV isolate was collected from is plotted on the x-axis, and the genome size in kpb is plotted on the y-axis. The circles are data points, whereas the line indicates the model fitted to all the data (equation 2). Panel B: results of model fitting, for all data or only the early outbreak data. The standard errors (SE) and confidence intervals (C.I.) of parameter estimates are given. In both analyses, *k* is significantly smaller than 1 as predicted by a geometric model of adaptation, where each new deletion occurring is smaller than the previous deletion.

We develop a simple mathematical model to describe the dynamics of WSSV genome shrinkage, and in particular to identify whether the rate of genome shrinkage is constant over time. We assume the genome size of ancestral virus introduced into aquaculture (*S_0_*) is a constant. We can then describe the evolution of genome size (*S*) as follows:
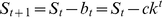
(1)where *t* is time (measured in years), *b_t_* is the deletion size at time *t*, *c* is the size of the first deletion *b_0_* and *k* is the multiplication factor for this initial deletion size. The multiplication factor *k* indicates whether the rate of genome shrinkage is constant over time. If *k* = 1 the rate of genome shrinkage is constant over time. If *k*>1 the rate of genome shrinkage increases over time. If *k*<1 the rate of genome shrinkage decreases over time. We can then calculate genome size at time *t* by subtracting the summation of the geometric sequence of all deletions which have occurred between time 0 and *t*-1 from genome size *S_0_*:
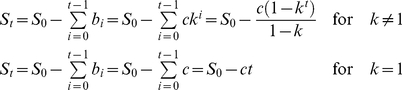
(2)To determine whether the rate of genome shrinkage was constant over time or not, we fitted the upper part of equation 2 to the observed genome size (see Materials and Methods), first for all the available data. The value for the multiplication factor *k* was significantly less than 1 (Fig. 4B). However, the data points from later outbreaks (‘first outbreak year’ >1996) might strongly influence the parameter estimates, due to the apparent attenuation of genome shrinkage. To avoid a bias towards a decreasing rate of genome shrinkage (*k* values <1), non-linear regression was repeated for the trajectory where total attenuation was not yet observed (i.e., the samples of the Philippines and Iran were removed). We refer to this subset as ‘early outbreak’ data. The value for the multiplication factor *k* was again significantly less than 1 (Fig. 4B). Both *k*-value estimates support a geometric model of genome shrinkage with the rate of shrinkage decreasing over time (Fig. 4B).

### Host survival and median host survival time of WSSV isolates

**Figure 5:**
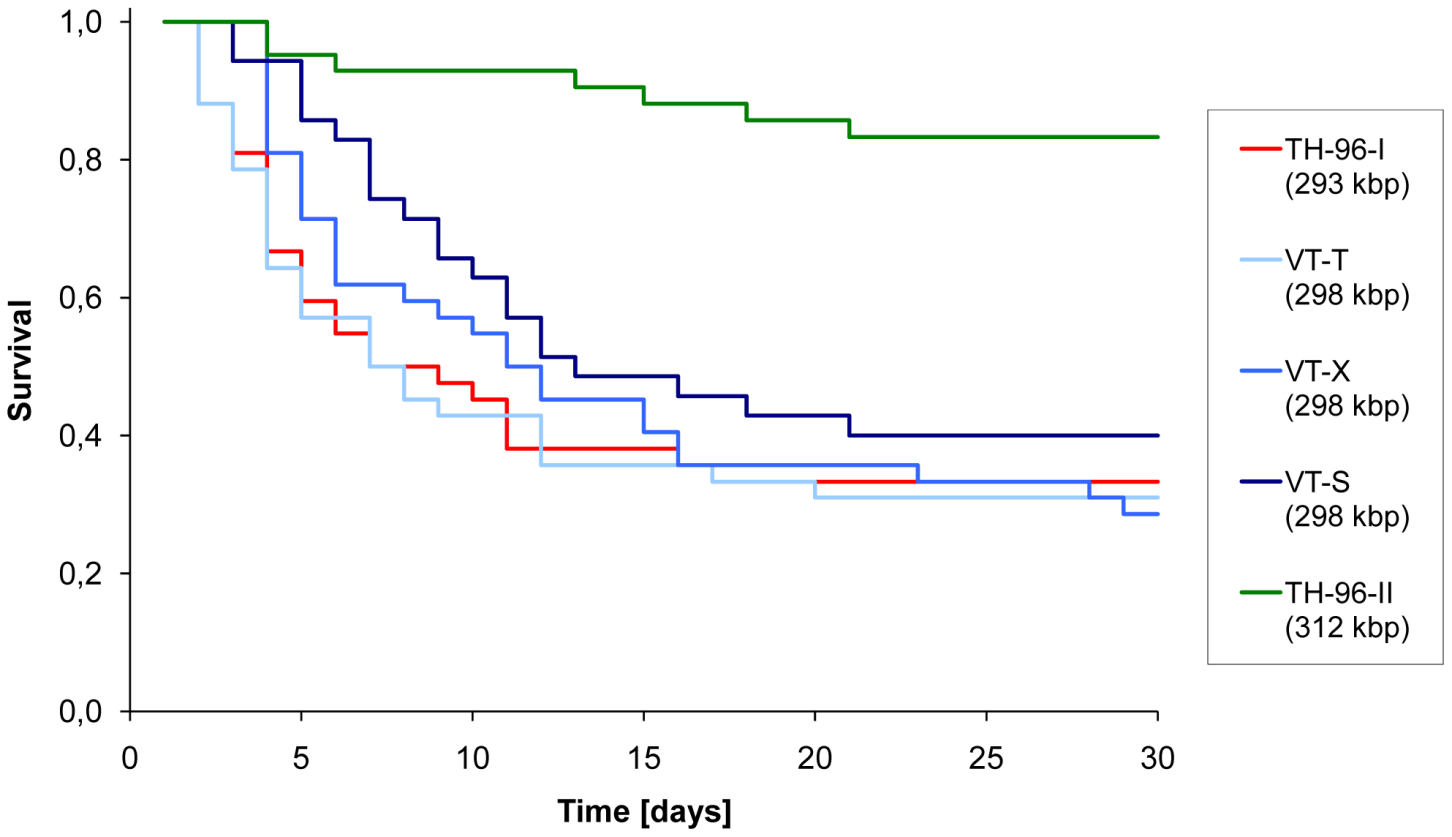
Kaplan-Meier survival curves for WSSV isolates with different genome sizes. Time in days is on the x-axis, and survival on the y-axis. The virus isolates and their genome sizes are given. Isolate TH-96-II has a significantly higher final survival, and a significantly longer median survival time than all other virus isolates when comparisons are made. There are no significant differences between the other virus isolates. The data are pooled from three different virus doses.

Given the contradictory previous reports on the relationship between genome size and fitness [8], [17], [19], we performed a bioassay to determine host survival (at the end of the experiment) and median host survival time for five WSSV isolates with different genome sizes: TH-96-II (312 kbp), VN-T, VN-X, VN-S (all 298 kbp), and TH-96-I (293 kbp) (Fig. 5). The proportion of host survival increased significantly with genome size (test for trend in proportions: χ2 = 21.32, *P*<0.001), supporting our hypothesis that fitness in an aquaculture environment increases as genome size decreases. When pair-wise comparisons were made, WSSV TH-96-II had a significantly higher survival than the other isolates (*P*<0.002 for all four comparisons), but there was no difference between TH-96-I and the Vietnamese isolates with intermediate deletions (P>0.05 for all three comparisons). A similar result was found for median survival times when making pair-wise comparisons with a log-rank test. Isolate TH-96-II had a significantly longer survival time than the other four isolates (*P*<0.01 for all comparisons), but there was no significant difference between TH-96-I and the Vietnamese isolates with intermediate deletions (*P*>0.05 for all three comparisons).

## Discussion

We genetically characterized WSSV isolates from five new Asian countries. For the two variable regions in which large genomic deletions occur – ORF14/15 and ORF23/24 – we found that the size of the deletions appears to stabilize over time. This observation suggests that there is an upper limit to deletion size at these loci, and that those genes or regulatory sequences up or downstream of the deletion are essential. ORF25 is an immediate early gene, whereas ORF13 and ORF16 are late genes, but no functions have been assigned [24]. We then tested whether there is a relationship between the size of the deletion at ORF14/15 and ORF23/24, and (i) ‘first outbreak year’ (the year of first outbreak in the country where the sample was collected), and (ii) the ‘distance from Taiwan’ (the ranked distance of the sample collection site from Taiwan). The ORF14/15 variable region was not significantly related to either independent variable (Table 2). The ORF23/24 variable region was only significantly related to ‘first outbreak year’, and not to ‘distance from Taiwan’ (Table 2). We previously found that the ORF23/24 variable region was also a suitable marker on a smaller spatiotemporal scale: WSSV spread within Vietnam [12].

Our results provide support for the hypothesis that WSSV molecular evolution at the ORF23/24 locus recapitulates the pattern of temporal spread, rather than being indicative of a smooth geographic radiation (Table 2). This outcome supports the view that commercial activities – such as the long-range transport of brood stock and post larvae – may have played an instrumental role in the spread of WSSV. This conclusion will be moot for WSSV given it is endemic in most shrimp producing countries. However, our analysis suggests that intervention strategies for other shrimp diseases should sufficiently focus on long-range transport. For example, strict measures were in place in the Philippines to prevent the entrance of WSSV, preventing establishment of the virus until 1999 [25]. These measures included a prohibition on the import of all exotic shrimp species and regulation of the within-country movement of shrimp fry.

The virus isolates genetically characterized here allow us to investigate the evolutionary trajectory of WSSV genome shrinkage for the first time. As these virus isolates were not collected at the time of first outbreak, we assume that the WSSV populations sampled were representative of the populations that first invaded a geographic region. The patterns found when retrospectively sampling WSSV populations suggest this approach is warranted [10], [12]. Others found that WSSV variants with intermediate-sized deletions were present in Southern China as late as 2007 [16]. Moreover, we have found that intermediate-sized deletions in the ORF23/24 variable region can be stably maintained in WSSV populations in extensive farms in Vietnam over many years [26]. We therefore think this approach is suitable, although it does raise some questions about the evolution of WSSV that deserve further consideration. Particularly, these observations imply that the geographic spread of WSSV is paired with rapid genome size evolution (i.e., the occurrence of progressively larger genomic deletions), whereas endemic populations are marked by stasis. We speculate that this may reflect the importance of within-host competitive fitness – and therefore strong selection for faster replication – during invasion of populations of naïve hosts. Once WSSV is endemic and many hosts are sub-lethally infected, fitness would be determined to a greater extent by longevity of infected hosts and other virus-host interactions. In other words, selection at the between-host level could – in an endemic situation – predominate over selection for faster replication at the within-host level (for examples of conflicting levels of selection see [27], [28]). Regional differences in WSSV virulence [29] and higher within-host competitive fitness of a WSSV variant with a small genome [8] are observations congruent with this hypothesis. Experimental quantification of transmission [30], [31] of geographical isolates under different conditions (e.g., host density, super-infection vs. co-infection) would provide a good test of this hypothesis.

Parameter estimates from our analysis of genome shrinkage support the hypothesis that the rate of genome shrinkage decreased over time (Fig. 4). It therefore appears as if the genome rapidly shrinks at first, but the rate of shrinkage decreases and eventually there is complete attenuation when the minimal genome size is reached. But why would genome shrinkage follow such a pattern? We propose two hypotheses: (i) an adaptive hypothesis and (ii) a neutral hypothesis.

**Figure 6:**
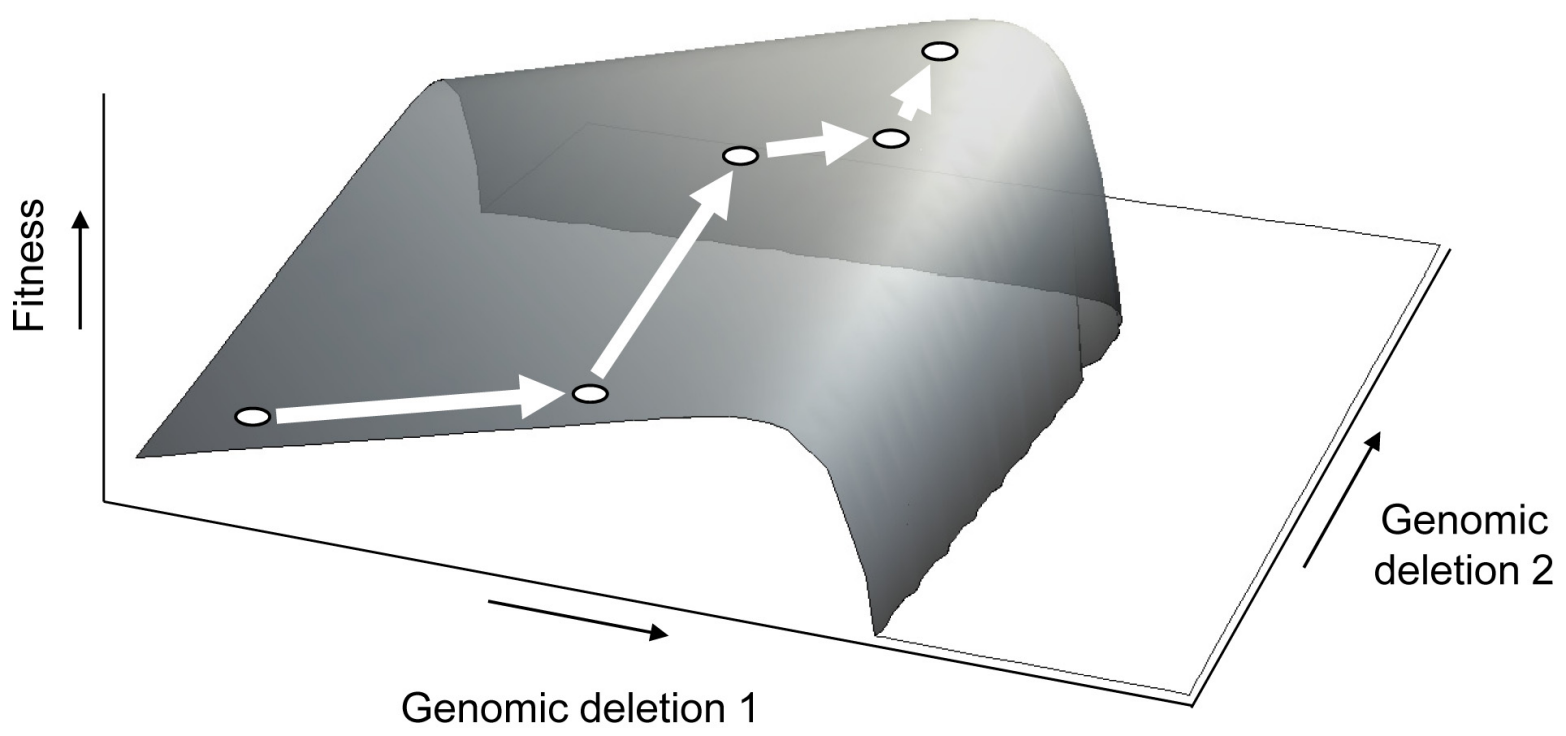
Conceptual fitness landscape for an organism evolving a smaller genome size. Arrows near the axes indicate the direction along which deletion size or fitness increases. Genomic deletions in two loci result in increased fitness, due to for example faster replication and reduced protein expression. However, if the genomic deletions become too large, non-redundant sequences are deleted and the organism may no longer be viable. The white arrows show a hypothetical example of our proposed model of evolution of genome size: initially large deletions occur, followed by ‘fine-tuning’ of genome size. Note that until both deletions approach the optimal size, there is no epistasis and the proposed fitness landscape is a flat, inclined plane.

Fisher proposed that an organism adapts to its environment by the substitution of mutations that slightly enhance fitness, because mutations resulting in small fitness changes are more likely to be beneficial than mutations causing large fitness changes [32]. Kimura then showed that mutations leading to larger fitness enhancement had a larger probability of becoming established, suggesting that mutations leading to intermediate fitness enhancement are most likely to be substituted [33]. More recently, theoretical work by Orr has shown that mean effects on fitness of the substituted mutations are similar to a geometric distribution, with each new mutation substituted in the population having a proportionally smaller effect on fitness than the previous mutation [22], [34], [35]. This pattern has been empirically observed for the evolution of fitness and morphological traits by experimental evolution [36], [37], [38], [39]. If WSSV genome size is linked to replicative fitness [8], [19], we can make use of this adaptive perspective to better understand the underlying dynamics. Mechanisms linking a smaller genome to increased fitness may be (i) the potential replicative advantages smaller genomes have over larger genomes, and (ii) reduced expression of redundant protein, leading to more efficient production of virions and hereby contributing to within-host competitive fitness. We then expect that the first substituted genomic deletion is large, and that subsequent deletions will be progressively smaller, until the optimum genome size is reached. A biological interpretation of this model is that those viruses with the smallest genome – but still retaining sufficient genomic sequences to replicate – are selected, at every time point in the evolutionary pathway. After initial selection for large deletions, there is fine-tuning of the genome size by ‘trimming away’ remaining redundant sequences flanking these large deletions (Fig. 6). Model parameter estimates from our data (Fig. 4B) support this model: the rate of genome shrinkage decreases over time (*k*<1). Our data suggest a rugged fitness landscape in the vicinity of optimum deletion size (Fig. 6), although this effect is less pronounced for ORF14/15 (Fig. 2) than ORF23/24 (Fig. 3); there appear to be clear limits to the size of these deletions.

Genome shrinkage is not necessarily an adaptive process. There is good evidence that population genetics in an altered environment (e.g., a change in effective population size) can drive genome shrinkage [40], [41]. An alternative hypothesis explaining patterns in WSSV genome shrinkage is therefore that these changes are neutral: the deletion of redundant genomic sequences [8], [11] would have no significant fitness benefit. Would the trajectory of genome shrinkage (Fig. 4) then be appreciably different than under an adaptive model? Although the rate of evolution under an adaptive model is faster, we speculate that the differences in the shape of the trajectory may be subtle. At the beginning of a neutral genome shrinkage process, there would be few constraints on what mutations could occur. Moreover, more distant loci would have a higher probability of homologous recombination, favoring the occurrence of large deletions. Later in the evolutionary pathway, there would be more constraints and smaller deletions would occur. To decide whether an adaptive or neutral model is best supported, we therefore consider whether there is evidence for a link between genome size and fitness.

The compact organization of the genome of many micro-organisms suggests that links between genome size and fitness exist [21], as has also been suggested for WSSV [8], [19]. Moreover, the ubiquity of defective interfering particles with small genomes and high rates of replication [42] suggests that replicative fitness and genome size are linked for many viruses. The link between genome size and fitness is contentious, however, both in our system [17] and others [20]. Nevertheless, the bioassay data we report here provide some support for this hypothesis (Fig. 5). The WSSV isolate with the largest genome size (Th-96-II, 312 kbp genome) induced a significantly higher level of host survival and a longer host survival time (Fig. 5). The ability of transmission stages to cause infection and host mortality – and therefore low host survival – is considered an important component of micro-parasite fitness, also in the case of WSSV [8]. In aquaculture conditions, the main route of WSSV transmission is via ingestion of infected cadaver material [31], [43], implying it is advantageous to kill the host quickly and hereby achieve earlier transmission than competitor strains. We therefore interpret low host survival and small median lethal times as indicators of high viral fitness in an aquaculture environment. However, there were no significant differences in the bioassay between viruses with intermediate genomes: VN-T, VN-X, VN-S (298 kbp), and the virus with a small genome TH-96-I (293 kbp) (see Fig. 5). One potential explanation for this observation is that the differences in fitness are too small to be detected. Direct competition experiments may be more sensitive to small fitness differences [8], [19]. However, if there is really no difference in fitness between these strains, the dynamics of genome shrinkage may be more complex than the adaptive model proposed. For example, we speculate that one of the deleted ORFs in the ORF14/15 or ORF23/24 variable regions may be deleterious for viral fitness in an aquaculture environment. Once this ORF would be deleted, further genomic shrinkage could be neutral.

One limitation to our bioassay is that we used natural virus isolates. Although most genomic variation is in the ORF14/15 and ORF23/24 variable regions [8], [10], we cannot exclude that other genomic variation contributed to the observed differences. Methods for generating recombinant strains [44] are not yet available for WSSV. We therefore included three WSSV isolates with a similar intermediate genome size (VN-T, VN-X and VN-S), but with variation in VNTR sequences [10]. This gave us an indication of whether other genomic variation plays a role in determining virulence. The results for these three genotypes were not significantly different (Fig. 5), providing support for the approach we took. A better experimental design would have been to use different isolates for the different genome sizes, but the necessary virus isolates were not available.

Up to two genetically characterized WSSV isolates per country were included for statistical tests on the ORF14/15 and ORF23/24 loci, and fitting the simple model of genome shrinkage (see Material and Methods). If multiple WSSV isolates from a single country could be included, then deletion size was never identical. We did not find this outcome surprising, however, because (i) we expect to find genetic variation on which selection and genetic drift can act in a rapidly evolving population, and (ii) we previously found variation between isolates collected in different regions of Vietnam [10]. Nonetheless, the deletion size was incongruent for the two Thai isolates included in the analysis (TH-S: 5785 bp deletion; TH-96-I: 13,210 bp; see Fig. 2). Thailand was at the time, however, the nexus of trade in shrimp products, broodstock and post-larvae [2], [45], [46]. We therefore speculate that WSSV was probably introduced into Thailand on many occasions, and we could therefore reasonably expect to observe high variation in Thailand. There is, however, another Thai isolate – collected soon after the isolates included in the analysis – without deletions in the ORF14/15 and ORF23/24 variable regions (TH-96-II; Fig. 2 and 3). We think this isolate is anomalous because (i) it contains sequences not found in any other WSSV isolate, and (ii) it was collected from shrimp which only developed disease symptoms after being imported to the Netherlands [8]. Unlike most other isolates included in our analyses, TH-96-II may, therefore, not be representative of WSSV strains causing outbreaks on farms at the time. This suggestion is supported by the low infectivity and within-host competitive fitness of this isolate [8], which imply that it probably would have been quickly displaced on shrimp farms. Finally, we fitted our model of genome shrinkage to a data set including TH-96-II. Similar parameter estimates were obtained, and estimated *k* values were significantly less than one (data not shown). The variation found in Thai isolates therefore does not affect the overall outcome of our analysis.

To our knowledge, we are the first to report the evolutionary trajectory during the shrinkage of a viral genome. Others have suggested an adaptive trajectory of incrementally smaller deletions may apply to genome shrinkage in bacteria [47], [48], [49]. This topic should receive further consideration, as there are many conceivable scenarios in which genome size will be under strong selection and show rapid evolution. Three pertinent cases are (i) emerging infectious disease outbreaks, as discussed here, (ii) the evolution of defective interfering particles [42], [50], [51], and (iii) evolution of genome size following genome duplication [52]. Experimental evolution [49], [53] is an excellent tool to further study the dynamics of genome shrinkage.

## Materials and Methods

### Ethics Statement

All animals were handled in strict accordance with good animal practice as defined by the relevant national animal welfare bodies.

### Collection of WSSV isolates and analysis of variable loci

The origins of the WSSV isolates analyzed are given in Table 1 and Fig. 1. Shrimp were cleaned with 70% ethanol and kept frozen during transportation to Wageningen University, The Netherlands, where samples were stored at −20°C until further processing. DNA extracts of collected shrimp were screened for the presence of WSSV with specific primers for VP26 [10]. The five WSSV variable loci previously identified [11], variable number tandem repeat (VNTR) loci ORF75, ORF94 and ORF125, and variable regions ORF14/15 and ORF23/24, were characterized up to the nucleotide level as described [10]. PCR on WSSV variable loci was performed with 250ng viral DNA, using Taq DNA polymerase (Promega; Madison, WI). The specific primer sets, PCR conditions used and sizes of the PCR products are shown in Table S1 (supplementary material). PCR products were analyzed and sequenced according to published procedures [10].

### Statistical analysis of ORF14/15 and ORF23/24 variable region data

For statistical analysis and modeling of WSSV genome size evolution, we combined the samples characterized here with other published reports containing information on the ORF14/15 and ORF23/24 variable regions (Table 1). A maximum of 2 samples per country were included, so that none of the countries would heavily affect the outcome. If more samples were available (i.e., for India and Vietnam), we chose samples that were collected earliest. WSSV TH-96-II was not included in this analysis because this sample is the putative common ancestor (archetype) for virus strains in shrimp aquaculture [8].

For the ORF14/15 and ORF23/24 variable regions, the non-parametric Jonckheere-Terpstra test [54] (SPSS 15.0, SPSS Inc., Chicago, IL) was used to determine if median deletion size significantly increased or decreased when the samples were ordered according to: (i) the year of first outbreak in the country where the sample was collected (“first outbreak year”), or (ii) the ranked geographic distance of the sample collection site to Taiwan, the location of the first WSSV outbreak (“distance”). As we are performing two comparisons for each locus, a Bonferroni correction [55] was therefore made to the significance threshold α so that the corrected threshold value (α′) is 0.025.

In order to determine ‘year of first outbreak’, we used a published list of reported outbreak years [2]. The presence of WSSV in Vietnam was confirmed by PCR in 1997 [56], but the first outbreaks occurred in 1993 [57], [58] and we used this date for our analysis. Cambodia is not included in the list of outbreak data we used [2]. Viral disease was implicated in the collapse of Cambodian shrimp aquaculture in the late 1990s, and we therefore took the year 1996, when shrimp production first declined [59], [60], as an estimate for the first outbreak.

### Model fitting

We fitted the upper part of equation 2 to the observed genome size (*S_obs,i_*), which was calculated as:
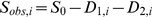
(3)where *S*_0_ is the genome size of the earliest WSSV isolate, WSSV-TW (So = 307,287 bp (Marks et al. 2004; Wang et al. 1995)), and *D*_1,*i*_ and *D*_2,*i*_ are the deletion sizes at ORF14/15 and ORF23/24 relative to the WSSV-TW sequence, for a virus isolate from a country in which the outbreak originated at time *i*, (number of years after 1992, thus *i* = 0 represents 1992). We only need to consider these two regions because the rest of the WSSV genome is surprisingly stable, with the exception of VNTR loci [8], [10], [11]. Non-linear regression (SPSS 15.0) was used to fit the upper part of equation 2 to the data and obtain 95% confidence intervals for the fitted constants *c* and *k*. Time of first outbreak was used as the independent variable in non-linear regression.

### Bioassay for host survival and median host survival time

Others have reported that WSSV replicative fitness is linked to genome size [8], [19]. To further detail these results, we used a bioassay to determine the level of host survival (at the end of the experiment) and median survival time of the following WSSV isolates: (i) TH-96-II: putative ancestral WSSV variant with a 312 kbp genome, the largest known WSSV genome [8], (ii) VT-T, VT-X and VT-S: Vietnamese isolates with an intermediate genome size of 298 kbp [10], and (iii) TH-96-I: a WSSV isolate collected early in the epizootic (1996) with small genome size of 293 kbp [4]. These five isolates were amplified in crayfish and virions purified as described elsewhere [61]. Subsequently, we determined the virion concentration for each purified virus stock by ELISA using IgY against WSSV-VP28 produced in bacteria, competitive PCR [62], and by counting intact virions with transmission electron microscopy. All methods gave similar results, and the ELISA data were then used to dilute all virus stocks to the same concentration (an absorbance of 0.445) using 330 mM NaCl buffer. 10^5^, 10^6^, and 10^7^ dilutions (in 330 mM NaCl) of these stocks were then used for the injection of shrimp.

The shrimp used for the experiment were SPF *Penaeus monodon* post larvae obtained from a commercial hatchery in Thailand, which we PCR screened for viral diseases [63]. The shrimp were communally kept at 28°C and fed commercial food pellets (Coppens International; Helmond, The Netherlands) prior to and during the experiment. Shrimp with a weight of 5–10 grams were intramuscularly injected with 10 µl dilutions of the WSSV stock using a 1.5 ml volume B–D Pen (Becton Dickinson) and 28Gx1/2″ NovoFine needles (Novo Nordisk). For each virus (5 isolates) and each dose (3 doses), 14 shrimp were injected. Shrimp were subsequently housed individually, and mortality was recorded daily. Non-injected control shrimp were kept, and no mortality was observed in these shrimp. Shrimp were maintained until 29 days post-injection. PCR with specific primers for VP26 [63] was performed to confirm deaths were due to WSSV infection, and randomly selected surviving shrimp were also screened for WSSV infection.

For analysis of host survival and median host survival times, we pooled data from all three doses to increase statistical power. Differences in the proportion of hosts surviving until the end of the experiment were made using a chi-squared test for trend in proportions (‘prop.trend.test’, R2.7.0, The R Foundation; Vienna, Austria). For this test the samples were ranked in order of increasing genome size, and pair-wise comparisons – with a Holm-Bonferroni correction – were also made (‘pairwise.prop.test’, R2.7.0). Median survival times were calculated from the estimated Kaplan-Meier survival curve, and the log-rank test was used to look for significant differences between treatments (SPSS 15.0).

## Supporting Information

Table S1 Table of primers used in PCR analysis.
(0.07 MB DOC)
