# Supplementary material for: Evolutionary Trajectory of White Spot Syndrome Virus (WSSV) Genome Shrinkage during Spread in Asia
Source: PLoS One. 2010 Oct 14;5(10):e13400. doi: 10.1371/journal.pone.0013400 (PMC2954812; doi:10.1371/journal.pone.0013400)
Supplement: Table S1 — Table of primers used in PCR analysis. (0.07 MB DOC) [file pone.0013400.s001.doc]

**Supplementary Material for Zwart et al.: Evolutionary Trajectory of WSSV Genome Shrinkage.**

**Table S1.** Primers used in PCR analysis for the variable loci of WSSV.

| **Primer pair name** | **Primer orientation** | **Sequence (5’-3’)** | **Annealing temperature (^0^C) / elongation time (s)** | **WSSV-CN sequence coordinates** | **Size (bp) of PCR product** |
| --- | --- | --- | --- | --- | --- |
| VR23/24 -Ja | Forward | CAGATAATGCAAACACGAGACAC | 49 / 100 | 275794-275816 | ~700 |
|  | Reverse | GTAAGTTTATTGCTGAGAAG |  | 286105-286086 |  |
| VR23/24 – Asia screen | Forward | GAGTAGTCTTCAATGGCAATGT | 55 / 80 | 275008-275029 | ~400 |
|  | Reverse | GATGACTCGGTACGCTTTAG |  | 287376-287357 |  |
| VR14/15-screen | Forward | GAGATGCGAACCACTAAAAG | 49 / 75 | 22904-22923^†^ | ~500/600 |
|  | Reverse | ATGGAGGCGAGACTTGC |  | 24157-24141^†^ |  |
| VR14/15-TH | Forward | GAGATGCGAACCACTAAAAG | 49 / 80 | 22904-22923^†^ | ~750 |
|  | Reverse | GAAAAATAAATCACGGGCTAATC |  | 23646-23624^†^ |  |

^†^ According to WSSV-TH-I-96 sequence
